# Enhanced Survival of Rifampin- and Streptomycin-Resistant Escherichia coli Inside Macrophages

**DOI:** 10.1128/AAC.00624-16

**Published:** 2016-06-20

**Authors:** Paulo Durão, Daniela Gülereşi, João Proença, Isabel Gordo

**Affiliations:** Instituto Gulbenkian de Ciência, Oeiras, Portugal

## Abstract

The evolution of multiple-antibiotic-resistant bacteria is an increasing global problem. Even though mutations causing resistance usually incur a fitness cost in the absence of antibiotics, the magnitude of such costs varies across environments and genomic backgrounds. We studied how the combination of mutations that confer resistance to rifampin (Rif^r^) and streptomycin (Str^r^) affects the fitness of Escherichia coli when it interacts with cells from the immune system, i.e., macrophages (Mϕs). We found that 13 Rif^r^ Str^r^ doubly resistant genotypes, of the 16 tested, show a survival advantage inside Mϕs, indicating that double resistance can be highly beneficial in this environment. Our results suggest that there are multiple paths to acquire multiple-drug resistance in this context, i.e., if a clone carrying Rif^r^ allele H526 or S531 acquires a second mutation conferring Str^r^, the resulting double mutant has a high probability of showing increased survival inside Mϕs. On the other hand, we found two cases of sign epistasis between mutations, leading to a significant decrease in bacterial survival. Remarkably, infection of Mϕs with one of these combinations, K88R+H526Y, resulted in an altered pattern of gene expression in the infected Mϕs. This indicates that the fitness effects of resistance may depend on the pattern of gene expression of infected host cells. Notwithstanding the benefits of resistance found inside Mϕs, the Rif^r^ Str^r^ mutants have massive fitness costs when the bacteria divide outside Mϕs, indicating that the maintenance of double resistance may depend on the time spent within and outside phagocytic cells.

## INTRODUCTION

Antibiotic resistance in many pathogens has become a worldwide problem, incurring both loss of human lives and economic costs (1). Bacteria can acquire antibiotic resistance as a result of transfer and acquisition of new genetic material between individuals of the same or different species but also by chromosomal mutations, which alter existing proteins. For instance, resistance to rifampin (Rif^r^), a rifamicin, occurs due to mutations in the gene *rpoB* coding for the β-subunit of RNA polymerase, and resistance to streptomycin (Str^r^), an aminoglycosidase, occurs due to mutations in the gene *rpsL* coding for a ribosomal protein (2). These genetic targets for resistance are common across a wide range of bacterial species, including Escherichia coli, Pseudomonas aeruginosa, and Mycobacterium tuberculosis (2–4).

Mutations causing antibiotic resistance usually incur a fitness cost in the absence of antibiotics (5–7). However, the magnitude of such costs is known to vary with the environment (8, 9). Even though most resistances are deleterious in the absence of antibiotics, some can be beneficial. Remarkably, rifampin resistance can even be selected for in populations evolving without antibiotics (10). Furthermore, evidence is mounting that epistasis is widespread among resistance mutations (2, 11, 12), and the level of epistasis is also dependent on the environment (13). Given the strong effect of genotype-environment interactions on the fitness of both single and double resistances, it is important to determine the effects of resistance in environments that are relevant in the context of infection.

We studied the fitness effects of double resistance mutations (Rif^r^ and Str^r^), when E. coli encounters macrophages (Mϕs), as will happen in an infection. Mϕs are key players of the host's innate immune system by recognizing, engulfing and killing microorganisms, and thus an important selective pressure in the context of infection. Escherichia coli is both a commensal and a versatile pathogen, acting as a major cause of morbidity and mortality worldwide (14), and there is evidence that some types of pathogenic E. coli evolved from commensal strains (15, 16). *E. coli* colonizes the infant gastrointestinal tract within hours after birth and typically builds a mutualistic relation with its host. However, it can become pathogenic when the gastrointestinal barrier is disrupted, as well as in immunosuppressed hosts (17–19). Nonpathogenic E. coli does not replicate inside Mϕs, but different mutants may have different abilities that persist inside these phagocytic cells (20). In a previous study, we found that E. coli clones with single point mutations in the *rpsL* gene, conferring Str^r^, exhibited a survival advantage over nonresistant E. coli in the intracellular niche of Mϕs (20). To determine whether such advantage would be altered in the presence of other resistances, we studied doubly resistant clones. We combined Str^r^ mutations—K43N, K43T, K43R, and K88R—with mutations that confer Rif^r^ and measured the competitive fitness of the double-resistance bacteria against a sensitive strain both inside and outside Mϕs. The chosen *rpoB* mutations conferring Rif^r^—S512F, S531F, H526Y, and I572F—exhibited variable effects in competition against sensitive clones (20). Mutations S512F and I572F showed a survival advantage inside Mϕs, S531F was neutral, and the H526Y phenotype was time dependent, being neutral at 2 h and beneficial at 24 h postinfection (20). Previous work (2, 11, 13, 21–23) has found strong epistatic interactions between alleles that confer rifampin and streptomycin resistance in different species and in different environments, a result with important consequences for understanding the possible evolutionary paths toward the acquisition of multiantibiotic resistance. Thus, we sought to answer the following questions. What are the fitness effects of Rif^r^ and Str^r^ when bacteria face pressure imposed by Mϕs? Does the survival advantage conferred by a single Str^r^ mutation depend on the presence of a Rif^r^ allele? Finally, do Mϕs show alterations in gene expression when infected with Rif^r^ Str^r^ mutants?

## MATERIALS AND METHODS

### Strains and media.

The RAW 264.7 murine macrophage cell line was maintained in an atmosphere containing 5% CO_2_ at 37°C in RPMI 1640 (RPMI; Gibco) supplemented with 2 mM l-glutamine (Gibco), 1 mM sodium pyruvate (Gibco), 10 mM HEPES (Gibco), and 50 μM 2-mercaptoethanol solution (Gibco), along with 10% heat-inactivated fetal bovine serum (Gibco). Bacterial strains were grown and competed in antibiotic-free RPMI medium in an atmosphere containing 5% CO_2_ at 37°C.

### Construction of strains.

We used susceptible E. coli K-12 MG1655 Δ*lacIZYA galK*::CFP/YFP strains and a collection of single Str^r^ and Rif^r^ mutants (also Δ*lacIZYA* *galK*::CFP/YFP) previously studied (2, 20). To construct the double Rif^r^ Str^r^ mutants, Rif^r^ and Str^r^ mutants were transferred into a background of each of the single Str^r^ and Rif^r^ mutants (Δ*lacIZYA* *galK*::CFP/YFP) by general transduction using P1 bacteriophage (24). To confirm the double mutations, each antibiotic resistance target gene was amplified by PCR and then sequenced. Each confirmed double-resistance clone was grown from a single colony in Luria-Bertani (LB) medium supplemented with the respective antibiotics and stored in 15% glycerol at −80°C.

### Survival assays inside the Mϕs.

**FIG 1:**
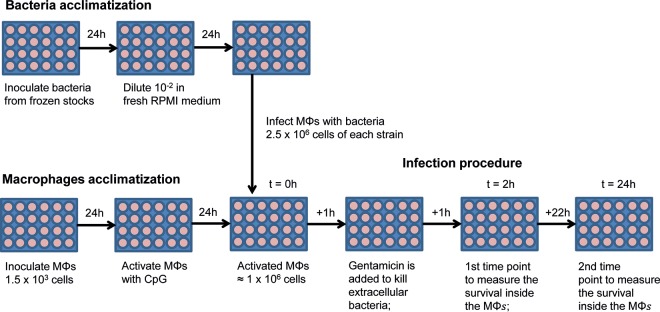
Experimental setup. Bacteria and macrophages were acclimatized independently for a total of 48 h. Macrophages were activated with CpG for 24 h during the period of acclimatization. After the period of acclimatization, 1 × 10^6^ macrophages were infected with 5 × 10^6^ bacteria (in a ratio of 1:1, resistant versus susceptible strain) labeled either with YFP or CFP. After 1 h of infection, the Mϕs were washed from the extracellular bacteria, and fresh RPMI cell culture medium containing 100 μg of gentamicin/ml was added to kill the remaining extracellular bacteria. To determine the number of intracellular bacteria after 2 and 24 h of incubation, infected Mϕs were washed with PBS plus 0.1% Triton-X in order to lyse the Mϕs. The overall number of bacteria was counted by plating them on LB agar plates. Survival inside the Mϕs was estimated as the change in relative frequency (Δ*X*), calculated as described in Materials and Methods.

To estimate the effect of double resistance on bacterial survival inside phagocytic cells, Mϕs were first seeded in plates for 24 h for acclimatization and then activated with 2 μg of CpG-ODN 1826 (5′-TCCATGACGTTCCTGACGTT-3′)/ml for 24 h (see Fig. 1). Afterward, the cells were washed from the remaining CpG-ODN, fresh antibiotic-free RPMI medium was added, and the Mϕs were infected with 5 × 10^6^ bacteria (at a 1:1 double-resistance/susceptible strain ratio) and centrifuged at 203 × *g* (1,000 rpm) for 5 min to enhance the bacterial internalization. The initial ratios of resistant and susceptible strains were determined by flow cytometry (see below). At 1 h of infection, the Mϕs were washed from the extracellular bacteria, and fresh cell culture medium containing 100 μg of gentamicin/ml was added to kill the remaining extracellular bacteria. To determine the number of intracellular bacteria after 2 and 24 h of incubation, infected Mϕs were washed with phosphate-buffered saline (PBS), and 0.1% Triton-X was added for 10 min at 37°C in order to lyse the Mϕs. The Mϕs were then centrifuged at 10,600 × *g* (10,000 rpm) for 5 min and washed in phosphate-buffered saline (PBS), and the overall number of bacteria was counted by plating on LB agar plates. Survival inside the Mϕs was estimated as the change in frequency (Δ*X*), measured as differences in viable cell counts, of the resistant strain, calculated as follows: Δ*X* = *Nf_b_*/(*Nf_a_ + Nf_b_*) − *Ni_b_*/(*Ni_a_ + Ni_b_*), where *Nf_a_* and *Nf_b_* are the numbers of resistant (*b*) and susceptible (*a*) bacteria after competition, and *Ni_a_* and *Ni_b_* are the initial numbers of resistant (*b*) and susceptible (*a*) bacteria before the competition. Significance was determined using a Wilcoxon signed-rank test.

### RNA extraction, reverse transcription, and quantitative real-time PCR (RT-qPCR).

To determine changes in macrophage gene expression after infection with bacteria, Mϕs (5 × 10^6^) were seeded per 6-well plate and infected independently (not in competition) with the chosen bacterial strain. Mϕs were treated as described above for the survival assays inside the Mϕs. At 2 h postinfection, the Mϕs were repeatedly washed with warm (37°C) RPMI prior to RNA extraction. RNA extraction was performed using a Direct-Zol RNA miniprep kit (Zymo Research) according to manufacturer's specifications. RNA was treated with RQ1 DNase (Promega) according to manufacturer's protocol. A reverse transcriptase reaction was performed with M-MLV RT (Promega) using random primers (Promega) according to manufacturer's protocol.

qPCR was executed in Bio-Rad CFX 384 with iTaq Universal SYBR green Supermix (Bio-Rad). Mϕ cDNA was diluted 10-fold before being used for qPCR. The cycling conditions were as follows: one step of 5 min at 95°C and then 40 cycles of 30 s at 95°C and 30 s at 60°C, and finally 30 s at 72°C. The primers used are listed in Table S1 in the supplemental material. Melting-curve analysis was performed to verify product homogeneity. All reactions included at least three biological replicates for each sample.

For analysis, data were normalized by the Pfaffl method (25) using the *actinB* housekeeping gene as reference for murine cDNA. When we compared the antibiotic resistance strains to the susceptible strain, the significant differences in expression levels were determined by a Student *t* test on the fold change values. Multiple *t* tests were performed to compare directly the double mutants K88R+H526Y and K88R+I572F.

### Competitive fitness in the presence and absence of Mϕs.

The double-resistant mutants constructed in the MG1655-CFP strain were competed against a susceptible MG1655-YFP strain in an antibiotic-free environment at a ratio of 1:1 under two different conditions in the presence or absence of Mϕs. Before the competitions, resistant and susceptible strains were grown separately in antibiotic-free RPMI medium for 48 h (with a dilution of 1:100 after 24 h) for acclimatization at 37°C with 5% CO_2_. For competitions in the presence of the Mϕs, 10^6^ Mϕs were seeded in the wells. Competitions in the presence or absence of Mϕs were then performed in 24-well cell culture tissue plates (containing 500 μl of RPMI culture medium in each well) by inoculating a mix of 2.5 × 10^4^ of each bacterial strain. The initial ratios of resistant and susceptible strains were determined by flow cytometry (see below). To determine the number of extracellular bacteria after 24 h of incubation, supernatant RPMI was diluted in PBS, and the overall number of bacteria was counted by plating the bacteria on LB agar plates. Competitive fitness outside the Mϕs was estimated as the change in relative frequency (Δ*X*), which was calculated as described above.

Significance for the competitive assays was determined using the Wilcoxon signed-rank test. A Wilcoxon rank-sum test was performed to analyze the behavior of the mutants in the presence or absence of Mϕs during the competitive fitness assessment. To test for a possible trade-off between competitive fitness in RPMI and survival inside Mϕs, a sign-test was used.

### Flow cytometry.

To determine the initial ratios of resistant and susceptible strains in the survival and competition assays, bacteria were quantified prior to infection with an LSR Fortessa flow cytometer using a 96-well plate autosampler. Samples were always run in the presence of SPHERO (AccuCount 2.0-μm blank particles) in order to accurately quantify bacterial numbers in the cultures. Briefly, flow cytometry samples consisted of 180 μl of PBS, 10 μl of SPHERO beads, and 10 μl of a 100-fold dilution of the bacterial culture in PBS. The bacterial concentration was calculated based on the known number of beads added. Cyan fluorescent protein (CFP) was excited with a 442-nm laser and measured with a 470/20-nm pass filter. Yellow fluorescent protein (YFP) was excited using a 488-nm laser and measured using a 530/30-nm pass filter.

## RESULTS

### Survival advantage of double resistance strains when competing inside Mϕs.

**FIG 2:**
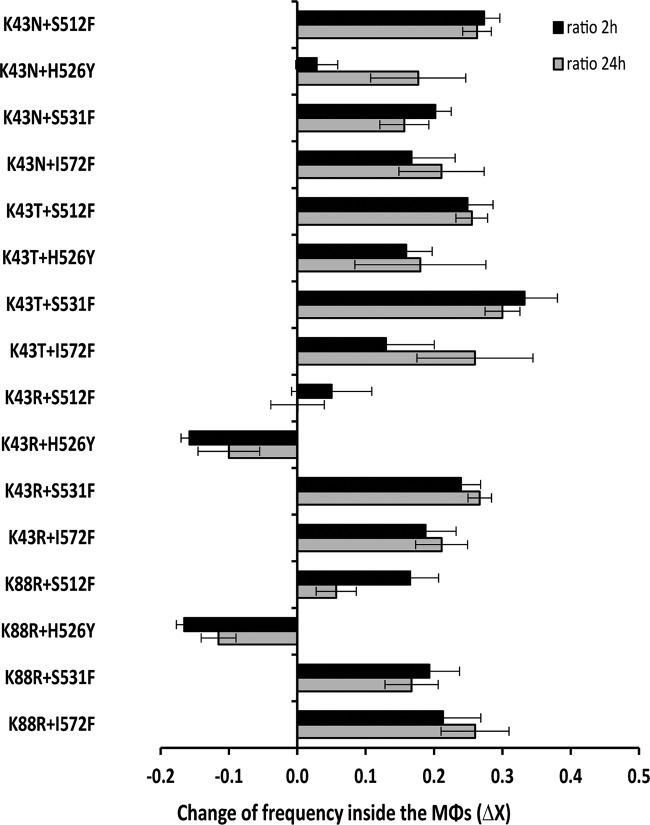
Rif^r^ Str^r^ double mutants display increased survival inside Mϕs. The graph shows the fitness effects of double antibiotic resistance on survival inside Mϕs at 2 h (■) and 24 h (▩) postinfection. All fitness effects were estimated using competition assays against a susceptible strain. At least five biological replicates were made for each measurement. All mutants showed statistical significance increases in frequency (*P* < 0.05, Wilcoxon signed-rank test) compared to the susceptible strain, except for K43R+S512F (at both 2 and 24 h postinfection), K88R+S512F (at 24 h postinfection), and K43N+H526Y (at 2 h postinfection). The results show that most Rif^r^ Str^r^ double mutants display an increased survival inside Mϕs. The opposite scenario occurs for two combinations which display sign epistasis: K43R+H526Y and K88R+H526Y.

Nonpathogenic E. coli K-12 does not replicate inside Mϕs, so survival is an important fitness component in this niche (20, 26). Survival inside the Mϕs was estimated as the change in frequency (Δ*X*), measured as differences in viable cell counts. We measured the relative survival ability of 16 E. coli K-12 strains carrying resistance to two antibiotics inside RAW 264.7 murine Mϕs. After growing double resistant and susceptible strains separately, we infected activated Mϕs in antibiotic-free medium with a coculture of bacteria. This coculture was obtained by mixing the appropriate volumes of resistant and susceptible strains so that they start competing at equal densities (one double resistant cell to one susceptible cell) in the coculture (Fig. 1). After 1 h of infection, gentamicin was added to kill the remaining extracellular bacteria, which is sensitive to this drug. To control for the efficacy of the gentamicin treatment, we plated the supernatant with bacteria, which were exposed 1 h to gentamicin, and detected a residual number of colonies of <10^3^ CFU/ml, which corresponds to <1% of the total numbers of bacteria found inside the Mϕs at the same time point (>10^5^ CFU/ml). To determine the relative numbers of resistant versus susceptible intracellular bacteria, infection was halted after 2 and 24 h of incubation, and the content of Mϕs was plated onto LB plates. We found that 13 of 16 double mutants showed a survival advantage inside Mϕs at either 2 or 24 h postinfection (Fig. 2**).** At 2 h postinfection, 62.5% of the double mutants displayed a significant increase in survival inside Mϕs, and this percentage increased to 81.3% at 24 h postinfection. These results indicate that the combination of Str^r^ Rif^r^ double resistance is generally beneficial inside Mϕs in the absence of antibiotics. All but one of the Rif^r^ Str^r^ double mutants resulting from combining any single (beneficial) Str^r^ mutation with beneficial Rif^r^ (S512F or I572F) showed increased survival inside the Mϕs compared to a susceptible strain. Thus, the combination of two resistances which individually are beneficial often results in an overall benefit for the double mutant. Two interesting cases of the opposite scenario were found. In the K43R+H526Y and K88R+H526Y combinations of double resistance, a decreased survival was observed even though each mutation alone does not confer a survival cost; these are examples of sign epistasis. By combining the results of the fitness effects of double resistance with the previously measured for single resistances (20), it follows as an outcome that single Rif^r^ mutations can acquire increased survival inside the macrophages by acquiring an Str^r^ mutation in 50% of the cases (see Fig. S1 in the supplemental material). For instance, the clinically common Rif^r^ S531F mutation, which is neutral when alone, may hitchhike with beneficial Str^r^ mutations, suggesting a path toward acquired double antibiotic resistance in the context of infection in the absence of antibiotics. To further corroborate this hypothesis, we performed competitions between the Rif^r^ Str^r^ double-mutant K43T+S531F against the single-mutant S531F (Rif^r^) and found that the double mutant outcompeted the single mutan inside the Mϕs (Δ*X* = 0.02 ± 0.01, *P* < 0.05). On the other hand, single Str^r^ mutations acquired increased survival inside the macrophages by acquiring a Rif^r^ mutation in 4 of 16 (25%) of the cases (see Fig. S1 in the supplemental material). The four combinations are K43N+S512F, K43T+S531F, K43R+S531F, and K88R+I572F.

### Double resistance showing sign epistasis prompts an altered inflammatory response.

**FIG 3:**
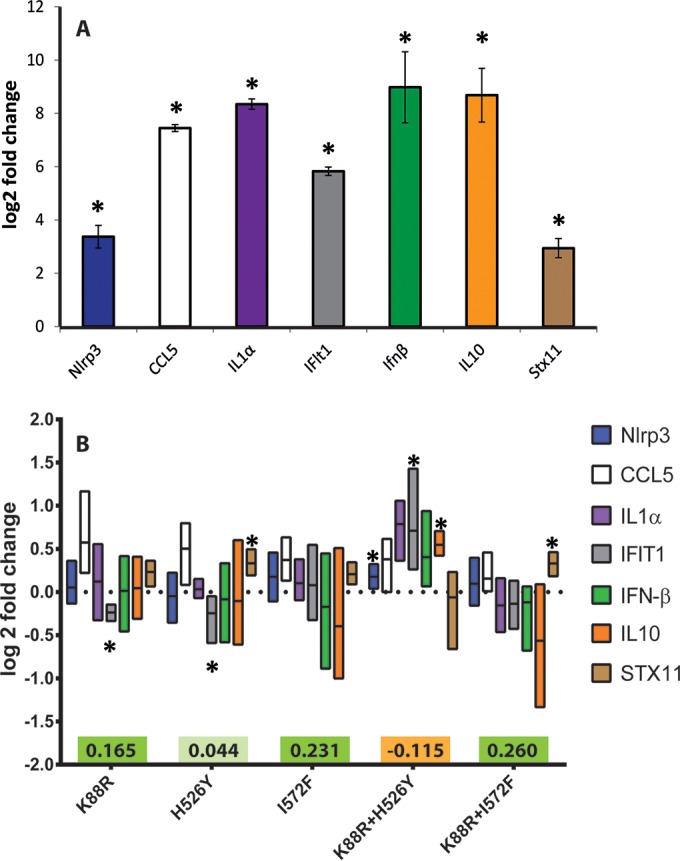
Double resistance with sign epistasis is associated with an enhanced proinflammatory response. (A) Relative amounts of murine transcripts of macrophages infected with E. coli MG1655 susceptible to antibiotics relative to transcript levels of uninfected macrophages (mock). The significant higher transcript levels of all the tested genes after infection evidence their role in this context (*P* < 0.01, one-sample *t* test). (B) Overall analysis by RT-qPCR of macrophage transcripts infected with different E. coli antibiotic-resistant mutants. The colored boxes show the survival effect (Δ*X*) of the mutants at 24 h postinfection. Data were normalized against a susceptible strain and are shown as the log_2_-fold change. At least three biological replicates were performed for each measurement.

Macrophages undergo changes in gene expression after the phagocytosis of bacteria (27). Given the differential survival of the double-resistant strains, we hypothesized that Mϕs gene expression could differ between the Rif^r^ Str^r^ mutants and the susceptible strain. We selected seven macrophage transcripts (*ccl5*, *ifit1*, *ifnβ*, *il1a*, *il10*, *nlrp3*, and *stx11*) previously identified as important in the context of bacterial infection (27) and tested their expression by RT-qPCR. In a previous work, we adapted E. coli to Mϕs by propagating bacterial populations for 30 days when facing Mϕs, while inhabiting both the intracellular and the extracellular environments (28). Infection of Mϕs with these E. coli strains previously adapted to Mϕs also led to an alteration in the expression of the tested genes (unpublished data). To confirm that all macrophage genes tested were significantly upregulated when bacterial infection occurs, we infected Mϕs with a susceptible strain and compared the transcription levels of the above-mentioned genes to those in a mock-infection experiment (i.e., uninfected Mϕs) (Fig. 3A). Having found that these genes were induced upon infection with the susceptible strain, we used the same set of genes to compare the transcriptional response by RT-qPCR of Mϕs infected by a susceptible strain or by several resistance strains. The Mϕs were infected independently but in parallel with a similar number of various bacterial strains: (i) the double Rif^r^ Str^r^ mutant strain K88R+H526Y (which showed sign epistasis that resulted in decreased survival inside the Mϕs) or K88R+I572F (which showed increased survival inside the Mϕs), (ii) the susceptible strain, (iii) a single resistant RpsL^K88R^ Str^r^ mutant, and (iv) a RpoB^H526Y^ and a RpoB^I572F^ mutant, each conferring Rif^r^. Figure 3B shows that, at 2 h postinfection, the expression of tested genes was altered in all but one of the resistance strains. Interestingly, for the infection with the K88R+H526Y mutant, which showed a decreased survival, three transcripts were significantly upregulated (Fig. 3B), whereas for the other mutants fewer changes were detected. The infection with mutant K88R+H526Y resulted in a significant increase in *ifit1* expression (*P* = 0.026, one-sample *t* test), *il-10* (*P* = 0.0005), and *nlrp3* (*P* = 0.009) relative to infection with a susceptible strain. Upon comparing the transcript expression levels between the K88R+H562Y and K88R+I572F infections, we found significant differences for *ifit1* (*P* = 0.022, multiple *t* test), *il1-α* (*P* = 0.014), and *il-10* (*P* = 0.012). Differences in the levels of *ifnβ* transcripts (*P* = 0.062) and *stx11* (*P* = 0.056) between the double mutants were marginally significant (0.05 < *P* < 0.1).

### Trade-off between survival and competitive fitness outside the Mϕs.

**FIG 4:**
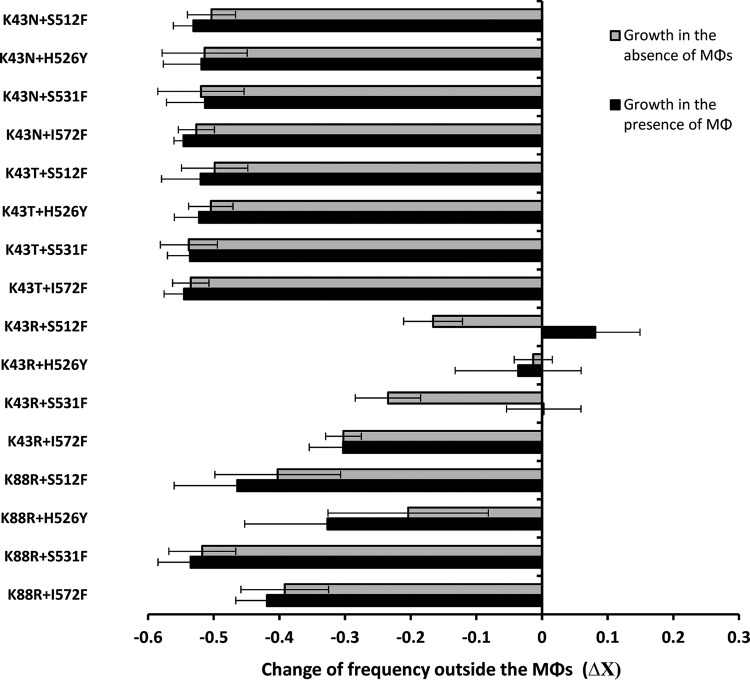
Trade-off between survival and competitive fitness outside Mϕs. The competitive fitness levels of Rif^r^ Str^r^ double mutants were measured in RPMI medium both in the absence (▩) and in the presence (■) of Mϕs. All fitness effects were estimated after 24 h using competition assays against a susceptible strain. At least three biological replicates were performed for each measurement. All mutants showed a statistically significant decrease in frequency (*P* < 0.05, Wilcoxon signed-rank test) compared to the susceptible strain except for K43R+S512F (in the presence of Mϕs), K43R+H526Y (in both the presence and the absence of Mϕs), and K43R+S531F (in the presence of Mϕs).

To determine the fitness effects of double resistance mutations when bacteria can grow outside macrophages, we performed competition assays (29) in two different environments: in RPMI medium alone (absence of Mϕs) or in RPMI medium with the presence of Mϕs (to which we did not apply gentamicin to allow for bacterial growth). Figure 4 shows that in most cases double resistance results in a strong decrease in competitive fitness in both environments. Remarkable exceptions were detected for the K43R+S512F, K43R+H526Y, and K43R+S531F double mutants, which show no competitive disadvantage when grown in the presence of Mϕs. The K43R+S512F mutant is a particularly worrisome combination of alleles, given that it results in a double-resistant clone with no fitness costs for survival inside Mϕs and a competitive growth advantage in the presence of Mϕs. However, a clear cost is measured when Mϕs where absent (*P* < 0.0001, Wilcoxon rank-sum test), which suggests that Mϕs are altering the medium to a more beneficial environment for this mutant. We have also found that K43R+H526Y is the only mutant that did not show a decreased competitive fitness when growing in RPMI, irrespective of the presence or absence of Mϕs (Fig. 4). This double mutant was actually one of the three exceptions that did not show increased survival inside the Mϕs at any of the time points measured. We noticed that the massive fitness costs observed for the Str^r^ Rif^r^ double mutants when bacteria are allowed to divide seemed to correlate with the substantial fitness benefits when bacteria are inside the Mϕs. Thus, we used our data for the Str^r^ Rif^r^ double mutants plus the available data from previous results for the single Str^r^ and Rif^r^ mutants (20) to test this hypothesis. We found a trade-off between survival inside the Mϕs and competitive fitness in RPMI both in the presence and in the absence of Mϕs (*P* < 0.01 in both cases [sign test]).

The observed loss in competitive ability of the double-resistance bacteria could be associated with a reduced nutritional competence (30, 31). To test for this, we analyzed the growth rates of the double Rif^r^ Str^r^ mutants by determining growth curves in RPMI under microaerobic conditions (without shaking). For all of the mutants, the growth curves displayed a biphasic behavior with two distinct growth rates separated by a short plateau (at an optical density at 600 nm of ≈0.4): an initial, higher growth rate (ε_r1_), presumably due to the presence of oxygen in small amounts in the RPMI medium, followed by a second lower growth rate (ε_r2_), presumably in the absence of oxygen (Table 1).

**TABLE 1:** Relative growth rates normalized to the susceptible strain

Rate and mutation	Mean relative growth rate (ε_r_) ± SEM			
	S512F	H526Y	S531F	I572F
ε_r1_				
K43N	0.240 ± 0.026	0.201 ± 0.008	0.114 ± 0.010	0.528 ± 0.221
K43T	0.187 ± 0.007	0.191 ± 0.002	0.241 ± 0.047	0.202 ± 0.005
K43R	0.203 ± 0.007	0.194 ± 0.003	0.203 ± 0.006	0.227 ± 0.005
K88R	0.194 ± 0.003	0.204 ± 0.021	0.157 ± 0.009	0.202 ± 0.009
ε_r2_				
K43N	0.772 ± 0.441	0.770 ± 0.053	0.666 ± 0.090	0.432 ± 0.027
K43T	0.420 ± 0.052	1.046 ± 0.232	1.008 ± 0.626	0.355 ± 0.020
K43R	0.843 ± 0.138	0.803 ± 0.133	0.758 ± 0.139	0.589 ± 0.039
K88R	0.643 ± 0.054	0.836 ± 0.146	0.579 ± 0.101	0.634 ± 0.118

## DISCUSSION

Multidrug-resistant bacteria pose a significant threat to human health, and it is important to determine the fitness effects of such bacteria during infection. Both single Str^r^ and Rif^r^ isolates have been identified in many important pathogens, such as Mycobacterium tuberculosis, Shigella flexneri, Vibrio cholerae, Pseudomonas aeruginosa, and even in commensal Escherichia coli sampled from healthy individuals (32–35). In the present study, we tested 16 Rif^r^ Str^r^ double mutants of E. coli for their ability to survive in the presence of Mϕs. This viability is an important fitness trait because numerous pathogens, which have evolved different mechanisms to survive inside the Mϕs, are rapidly acquiring multidrug resistance to these drugs. For instance, M. tuberculosis owes its success as pathogen to its ability to interfere with the normally effective antimicrobial properties of the macrophage and is frequently both Str^r^ and Rif^r^ (36–39). We found that most Rif^r^ Str^r^ mutants in E. coli had increased survival inside Mϕs after 24 h postinfection, and a similar effect was also observed at 2 h postinfection. It would be important to determine whether similar effects are true for the combinations of the highly frequent *rpoB*(*H526Y*) and *rpoB*(*S531L*) mutations in natural pathogens, such as M. tuberculosis (4, 38, 39). In fact, our E. coli results suggest that such pathogens could benefit from the combination of these Rif^r^ alleles with certain Str^r^ alleles and suggests a possible path to acquire multidrug resistance in the context of infection and in the absence of antibiotics. This finding suggests that streptomycin treatment should be avoided in patients infected with Rif^r^ mutants.

Our findings regarding the fitness benefits of Rif^r^ Str^r^ mutations in the absence of antibiotics add to the cases recently found for other resistances. For instance, it has been shown that knockouts of the *opr*D and *glp*T genes, resulting in antibiotic resistance to carbapenem and fosfomycin, also provided an *in vivo* fitness advantage during infection of P. aeruginosa in the absence of drugs (40, 41). In this same organism, the loss of genes such as *ampC* (encoding a cephalosporinase conferring resistance to amoxicillin-clavulanic acid), *aph* (encoding an aminoglycoside phosphotransferase conferring resistance to kanamycin), and the *mexAB-oprM* operon (encoding an efflux pump conferring resistance to both nalidixic acid and trimethoprim-sulfonamide) bears a fitness cost in the absence of antibiotics, indicating that these genes are important fitness determinants for both gastrointestinal colonization and lung infection (40) in the absence of antibiotics. Another study has shown that Staphylococcus aureus can acquire intermediate levels of resistance to vancomycin in the absence of antibiotic and during *in vivo* infection in a mouse model solely due to competition between coevolving bacterial strains (42). Overall, our results add to a growing body of evidence suggesting that a reduction in antibiotic use, which *a priori* should lead to a decrease in (multi)drug-resistant strains, might produce an unfortunate outcome, a finding that contrasts with the currently prevailing view that increased antibiotic resistance has a negative fitness cost.

In our sample of double resistance, we found two cases of sign epistasis for survival of the bacteria inside the Mϕs, where each single resistance is either beneficial or neutral, but the combination is deleterious. When we compared the expression level of genes in Mϕs infected with a double-resistant mutant exhibiting sign epistasis (K88R+H526Y), we found that several genes were upregulated. The significant upregulation of NLRP3 and IFIT1 (interleukin-1α [IL-1α] compared directly with the results obtained for the K88R+I572F) point to an exacerbated proinflammatory response from the Mϕs when in the presence of K88R+H526Y. Indeed, NLRP3 is activated in response to a variety of pathogen-associated and danger-associated molecular patterns, and the active NLRP3 inflammasome leads to the secretion of potent proinflammatory cytokines. Escherichia coli has previously been shown to induce NLRP3 activation in Mϕs (43, 44), and enterohemorrhagic E. coli is able to target NLRP3 inflammasome activation and block IL-1β cytokine production (45). It would be interesting to study the fitness effects of these resistances in this pathogenic strain. IFIT1 is induced upon treatment with interferon (IFN), in particular IFN-α/β, and is better characterized in the context of a viral infection (46). IFN-β is also involved in the regulation of NLRP3 inflammasome (47, 48). The observed upregulation of IL-1α, a protein involved in various immune responses and inflammatory processes, is also in agreement with a proinflammatory response from the Mϕs. These cytokines are produced by Mϕs in response to cell injury and are involved in the inflammatory response with many interactions with other cytokines, ultimately inducing apoptosis (49). On the other hand, we also saw a significant upregulation of *il10* (a 0.55-log_2_-fold change) in the presence of this double mutant. The protein encoded by *il10* is a cytokine produced primarily by monocytes with pleiotropic effects involved in limiting the inflammatory response (50). Together, our results suggest that K88R+H526Y mutant may be able to modify the inflammatory response by the Mϕs compared to the susceptible strain in the specific experimental conditions that we tested. In a real infection, both bacterial numbers and macrophage numbers are likely to be variable, so this effect may be dependent on the context. It is noteworthy to compare our results with those from a previous study by Mavromatis et al. (51), who performed a cotranscriptomics analysis in Mϕs infected with two phenotypically different uropathogenic E. coli strains, one able to survive and another unable to survive within Mϕs. Mavromatis et al. did not detect significant host gene expression differences after infection with the different bacterial strains at 2 and 4 h postinfection. Only one gene (*Slc7a11*) encoding a cysteine/glutamate exchanger was found to be upregulated at 24 h postinfection for the strain that was able to survive inside the Mϕs (51). In our bacterial strains, which only differ in the mutations conferring resistance to antibiotics, several Mϕ genes were found to be differently upregulated, especially in the double mutant that displayed sign epistasis.

Our results also suggest that the increased survival inside the Mϕs conferred by the double resistance is associated with a substantial loss of competitive fitness in RPMI. The results displayed in Table 1 also show that Rif^r^ Str^r^ double resistance incurs a strong cost in the initial growth rate (ε_r1_), but this cost is reduced along with growth. This finding is in agreement with the notion that the Rif^r^ Str^r^ mutants are less able to compete for the resources present in RPMI and is consistent with the observed decreased competitive fitness (Fig. 4).

Lower growth rates and increased survival suggest that antibiotic resistance mutations might be tilting the so-called SPANC balance (self-preservation and nutritional competence) to an increased general stress response and starvation survival at the expense of a decreased nutritional ability (30, 31). Mutations in the *rpsL* gene, conferring Str^r^, improve the accuracy of ribosomes but also slow down the translation process (52, 53), and slower ribosomes could explain the observed lower growth rates in RPMI. Concurrently, although fast ribosomes are required in actively dividing cells, hyperaccurate ribosomes are advantageous in nondividing cells because they lower the fraction of misfolded proteins, which are known to be more prone to protein oxidation during growth arrest (54). This should be extremely relevant upon entry to the Mϕs, where E. coli undergoes growth arrest and nutrient starvation. Importantly, the trade-off between survival and competitive fitness seems to be strong enough to prevent the dissemination of multiantibiotic resistance. However, while the E. coli K-12 strain used for this study is not able to replicate in the phagolysosome, many intracellular pathogens can replicate inside the macrophages (55). For pathogens that are mainly intracellular, it remains an open question how strong the described trade-off will be.

## Supplementary Material

Supplemental material
