# Supplementary material for: Enhanced Survival of Rifampin- and Streptomycin-Resistant Escherichia coli Inside Macrophages
Source: Antimicrob Agents Chemother. 2016 Jun 20;60(7):4324–32. doi: 10.1128/AAC.00624-16 (PMC4914683; doi:10.1128/AAC.00624-16)
Supplement: Supplemental material [file supp_60_7_4324__index.html]

Enhanced Survival of Rifampin- and Streptomycin-Resistant Escherichia coli Inside Macrophages — Supplemental material 

# Enhanced Survival of Rifampin- and Streptomycin-Resistant Escherichia coli Inside Macrophages

## Supplemental material

- Supplemental file 1 -

  Table S1 and Figure S1

  PDF, 250K
