# Supplementary material for: Enhanced Survival of Rifampin- and Streptomycin-Resistant Escherichia coli Inside Macrophages
Source: Antimicrob Agents Chemother. 2016 Jun 20;60(7):4324–32. doi: 10.1128/AAC.00624-16 (PMC4914683; doi:10.1128/AAC.00624-16)
Supplement: Supplemental material [file AAC.00624-16_zac007165336so1.pdf]

Supplemental Table 1

List of primers used for RT-qPCR experiment

Amplified region	Primers (5` - 3`)
**Actb**	F ATCGTGCGTGACATCAAAGA<br>R AAGGAAGGCTGGAAAAGAGC
**IFN1B**	F CACAGCCCTCTCCATCAACT<br>R GCATCTTCTCCGTCATCTCC
**IL10**	F CCAAGCCTTATCGGAAATGA<br>R TTTTCACAGGGGAGAAATCG
**NLRP3**	F ATGCTGCTTCGACATCTCCT<br>R AACCAATGCGAGATCCTGAC
**STX11**	F CAGGGCAAGTGGGATGTATT<br>R GTGTCCTCCTGCTTCTCCAC
**IFIt1**	F CCAAGTGTTCCAATGCTCCT<br>R GGATGGAATTGCCTGCTAGA
**Il1a**	F AGTCGGCAAAGAAATCAAGATG<br>R CCTTGAAGGTGAAGTTGGACA
**Ccl5**	F ATATGGCTCGGACACCACTC<br>R GTGACAAACACGACTGCAAGA

Supplemental Figure 1

ΔX 24h		S512F	H526Y	S531F	I572F
		0.350	0.044^{ns}	-0.013^{ns}	0.231
K43N	0.248	0.263	0.177	0.157	0.211
K43T	0.258	0.255	0.180	0.300	0.260
K43R	0.231	0.000^{ns}	-0.100	0.267	0.211
K88R	0.165	0.057^{ns}	-0.115	0.167	0.260

< -0.200
-0.150 to -0.200
-0.100 to -0.150
-0.050 to -0.100
-0.050 to +0.050
0.050 to 0.100
0.100 to 0.150
0.150 to 0.200
> 0.200

Supplemental Figure 1 - Observed survival inside the MΦs of single and double resistant clones at 24h post-infection measured as a change in the frequency of resistant bacteria (ΔX 24h) suggest pathways to acquire Rif and Str double resistance. For instance, the results show that Rif^R H526Y and S531F mutants often benefit from acquiring a Str^R mutation, in the absence of antibiotics, to adapt to the intra-macrophage environment. The data from the single resistances is taken from Miskinyte and Gordo, 2013 and the data for the double mutants were determined in this study.

Supplemental References

Miskinyte M, Gordo I. 2013. Increased survival of antibiotic-resistant *Escherichia coli* inside macrophages. Antimicrob Agents Chemother. **57**:189–195.
